# Correction to *Lancet Microbe* 2024; 5: e119–30

**DOI:** 10.1016/j.lanmic.2025.101309

**Published:** 2025-12

*Kim J W, Bowman K, Nazareth J, et al. PET-CT-guided characterisation of progressive, preclinical tuberculosis infection and its association with low-level circulating* Mycobacterium tuberculosis *DNA in household contacts in Leicester, UK: a prospective cohort study.* Lancet Microbe *2024;* ***5:*** *e119–30*—In this Article, additions have been made to the Methods to clarify that Actiphage testing was performed at the Royal Veterinary College and the University of Leicester following different procedures. Measures taken to minimise the risk of contamination were also included in the Methods. The Contributors section has been amended to detail the role of authors in Actiphage testing. Appendix 2 has been updated to correct some Actiphage Ct values (appendix 2 p 4), which did not affect the results or their interpretation. These corrections have been made as of Dec 11, 2025.

